# Supplementary material for: The interactome of intact mitochondria by cross-linking mass spectrometry provides evidence for coexisting respiratory supercomplexes
Source: Mol Cell Proteomics. 2017 Dec 8;17(2):216–32. doi: 10.1074/mcp.RA117.000470 (PMC5795388; doi:10.1074/mcp.RA117.000470)
Supplement: Supplemental Data [file supp_RA117.000470_133922_0_supp_23978_fzffwf.zip › spectra_annotation/mito_DR_spectra_annotation/393-1-4-1-8-1.pdf]

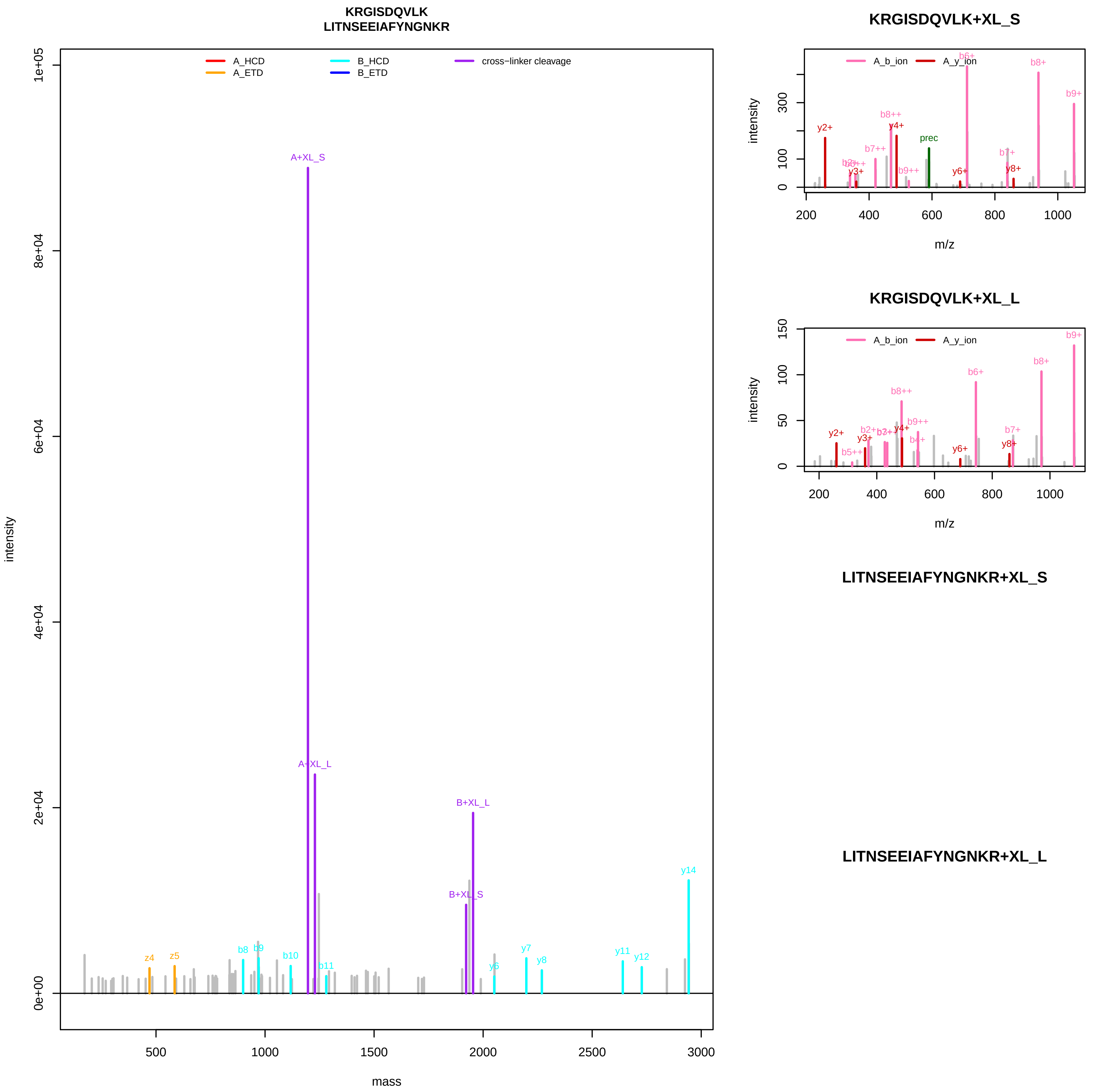
KRGISDQVLK
LITNSEEIAFYNGNKR
A_HCD
A_ETD
B_HCD
B_ETD
cross−linker cleavage
A+XL_S
A+XL_L
B+XL_L
B+XL_S
z4
z5
b8
b9
b10
b11
y6
y7
y8
y11
y12
y14
intensity
mass
0e+00
2e+04
4e+04
6e+04
8e+04
1e+05
500
1000
1500
2000
2500
3000
KRGISDQVLK+XL_S
A_b_ion
A_y_ion
prec
y2+
y3+
y4+
y6+
y8+
b7++
b8++
b9++
b6+
b7+
b8+
b9+
intensity
m/z
KRGISDQVLK+XL_L
A_b_ion
A_y_ion
y2+
y3+
y4+
y6+
y8+
b2+
b3++
b4+
b5++
b8++
b9++
b6+
b7+
b8+
b9+
intensity
m/z
LITNSEEIAFYNGNKR+XL_S
LITNSEEIAFYNGNKR+XL_L
